# *Vitex agnus castus* Extract Ze 440: Diterpene and Triterpene’s Interactions with Dopamine D2 Receptor

**DOI:** 10.3390/ijms252111456

**Published:** 2024-10-25

**Authors:** Jakob K. Reinhardt, Lukas Schertler, Hendrik Bussmann, Manuel Sellner, Martin Smiesko, Georg Boonen, Olivier Potterat, Matthias Hamburger, Veronika Butterweck

**Affiliations:** 1 Department of Pharmaceutical Sciences, Pharmaceutical Biology, University of Basel, Klingelbergstrasse 50, 4056 Basel, Switzerlandolivier.potterat@unibas.ch (O.P.); 2 Medical Department, Max Zeller Soehne AG, Seeblickstrasse 4, 8590 Romanshorn, Switzerlandhendrik.bussmann@zellerag.ch (H.B.); georg.boonen@zellerag.ch (G.B.); 3 Department of Pharmaceutical Sciences, Computational Pharmacy, University of Basel, Klingelbergstrasse 50, 4056 Basel, Switzerlandmartin.smiesko@unibas.ch (M.S.)

**Keywords:** *Vitex agnus castus*, microfractionation, D2 receptor, triterpene, diterpene, molecular docking

## Abstract

Pre-clinical studies suggest that extracts prepared from the fruits of *Vitex agnus castus* (VAC) interact with dopamine D2 receptors, leading to reduced prolactin secretion. In previous experiments, dopaminergic activity was mostly evaluated using radioligand binding assays or via the inhibition of prolactin release from rat pituitary cells. Diterpenes featuring a clerodadienol scaffold were identified as major active compounds, but no conclusive data regarding their potency and intrinsic activity are available. Utilising advances in chromatography, we re-examined this topic using HPLC-based tracking of bioactivity via microfractionation of the VAC extract Ze 440. Using a cAMP-based assay, we measured dopaminergic activity in CHO-K1 cells that overexpress the human D2 receptor. Six diterpenes were isolated from two active HPLC microfractions. Viteagnusin I emerged as the most potent diterpene (EC_50_: 6.6 µM), followed by rotundifuran (EC_50_: 12.8 µM), whereas vitexilactone was inactive (EC_50_: >50 µM). Interestingly, triterpenes were also identified as active, with 3-*epi*-maslinic acid being the most active compound (EC_50_: 5.1 µM). To better understand these interactions at the molecular level, selected diterpenes and triterpenes were analysed through molecular docking against D2 receptor structures. Our data show that the dopaminergic activity of VAC diterpenes seems to depend on the configuration and on ring substitution in the side chain. This study also highlights for the first time the dopaminergic contribution of triterpenes such as 3-epi-maslinic acid.

## 1. Introduction

Dopamine (DA) plays a crucial role in regulating the synthesis and secretion of prolactin (PRL). In fact, DA is a potent inhibitor of PRL secretion (prolactin inhibiting factor; PIF), which is achieved through direct interactions with D2 receptors present on the membranes of lactotrophic cells. The activation of these receptors leads to the downregulation of PRL gene expression via the inhibition of adenylyl cyclase and inositol phosphate metabolism. Furthermore, dopamine modifies various potassium and calcium channels, ultimately inhibiting PRL exocytosis. Hyperprolactinaemia and reproductive disorders are the result of disturbed hypothalamic dopamine function (for a review, see [1]).

The polypeptide PRL is mainly produced by lactotrophs in the anterior pituitary and is best known for its vital role in breast physiology and lactation. The secretion of PRL is regulated by dopamine via a negative feedback mechanism [2]. Abnormal secretion of PRL can lead to significant pathological consequences [3,4,5]. Maintaining an appropriate level of PRL is therefore crucial for normal lactation, and any imbalances can compromise this ability. Insufficient PRL levels prevent milk production in lactating females, while inappropriately elevated levels can cause galactorrhoea in non-breastfeeding females or males [3,4,5]. Moreover, disruptions in PRL balance can profoundly affect the menstrual cycle. It has been shown that excessive PRL secretion in females can lead to amenorrhea, as a result of the inhibition of gonadotropin-releasing hormone (GnRH) release by PRL [6]. Hyperprolactinemia is also an important factor in the aetiology of premenstrual syndrome (PMS) [7]. Increased levels of PRL have been measured in response to stressful events or before menstruation, leading to premenstrual mastodynia, water retention, depression, and/or dysphoria, as well as infertility. Furthermore, increased levels of PRL can decrease the development of the corpus luteum, thereby decreasing the production of progesterone [8].

Among women of reproductive age, PMS is one of the most prevalent disorders during the luteal phase of the menstrual cycle. It has been observed in multiple epidemiologic surveys that up to 20% of women in the age group between 30 and 40 years suffer from moderate to severe PMS symptoms [9]. Symptoms of PMS include, for example, mood swings, headache, weight gain due to water retention, premenstrual mastodynia, and insomnia [10]. For some women, the severity of symptoms interferes with daily life activities. Considering the high proportion of working-age women, the impact of PMS on productivity, especially in terms of absenteeism and inefficiency at work, is a significant economic factor [11,12].

A wide range of treatment options for PMS exist, including hormonal (e.g., progesterone) and non-hormonal (e.g., antidepressants) treatment options. Safer and less intense approaches to managing PMS symptoms include lifestyle changes and treatment with herbal medicines [13]. In this regard, extracts prepared from the fruits of chasteberry (*Vitex agnus castus*, VAC; family of Lamiaceae) are widely used to treat PMS symptoms [14,15,16,17]. Numerous studies have shown that VAC extracts exert dopaminergic activity by binding to D2 receptors, resulting in the inhibition of PRL secretion [18,19,20]. In this regard, it should be pointed out that VAC is not the only plant exerting PRL inhibitory effects. A decrease in plasma PRL levels, and thus dopaminergic activity, has been reported for *Echinacea purpurea*, *Hypericum perforatum*, and *Ginkgo biloba* [21,22,23]. Pilot studies in patients with PMS could show that *H. perforatum* and *G. biloba* could reduce PMS symptoms [24,25]. However, the compounds responsible for the dopaminergic effects of these plants still remain unknown.

Various diterpenes derived from a lipophilic fraction of a VAC extract have demonstrated dopaminergic properties and the ability to inhibit PRL secretion [18,19,20]. However, in these earlier studies, dopaminergic activity was primarily assessed through radioligand binding assays or by inhibiting PRL release from rat pituitary cells. Conclusive data regarding the potency of isolated diterpenes and their intrinsic activity remain lacking. Given the technological advancements in chromatography as well as in cell-based screening assays, it was the aim of the present study to revisit this topic using HPLC-based tracking of bioactivity via microfractionation [26,27,28] of the ethanolic VAC extract Ze 440. Furthermore, recent advances in structural biology methods allowed for the elucidation of the atomic structure of several ligand–dopamine D2 receptor complexes [29,30,31,32,33,34]. Several modelling studies have demonstrated that molecular docking-based approaches can be used to establish the structure–activity relationships of synthetic and natural compounds [35,36,37,38]. We thus investigated the binding of selected compounds using multiple established docking programs in combination with an ensemble of crystal and electron microscopic structures of the dopamine D2 receptor.

## 2. Results

### 2.1. Ze 440 VAC Extract and Different HPLC Microfractions Activate the D2 Receptor

**Figure 1:**
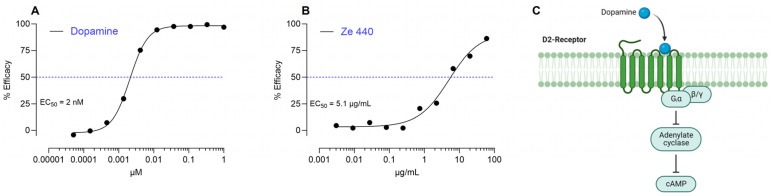
**Dopamine and Ze 440 whole VAC extract activate the D2 receptor.** Representative EC_50_ profiles of (**A**) dopamine and (**B**) VAC extract Ze 440 at the D2 receptor measured using a functional bioassay in CHO-K1 cells overexpressing the human D2 receptor (n = 2 independent experiments). Dots show the mean of technical triplicates. EC_50_ values were determined using Graphpad Prism 9.0. (**C**) Schematic illustration of dopamine binding to its D2 receptor. The image was created with BioRender.com (accessed on 10 November 2023).

Previous pharmacological studies have determined the dopaminergic activity of VAC extracts via D2 receptor radioligand binding assays or by measuring PRL levels in cell culture supernatants. In order to re-examine this topic using an HPLC activity profiling-based approach [26,28], we first validated our read-out system. Therefore, purified dopamine or Ze 440 (whole VAC extract) were evaluated for their ability to activate the D2 receptor in a cell-based bioassay by measuring cAMP levels (Figure 1A–C). The positive-control dopamine could activate the D2 receptor with an EC_50_ of 2 nM. In line with previous findings, the VAC extract Ze 440 was also found to activate the D2 receptor with an EC_50_ of 5.1 µg/mL (Figure 1B). Due to solubility issues, it was not possible to test concentrations above 60 µg/mL. It is worth mentioning that the findings described here cannot be extrapolated to other VAC extracts apart from Ze 440. In addition to the proprietary extraction conditions, Ze 440 is produced using VAC fruits of the Vitan variety only [39].

**Figure 2:**
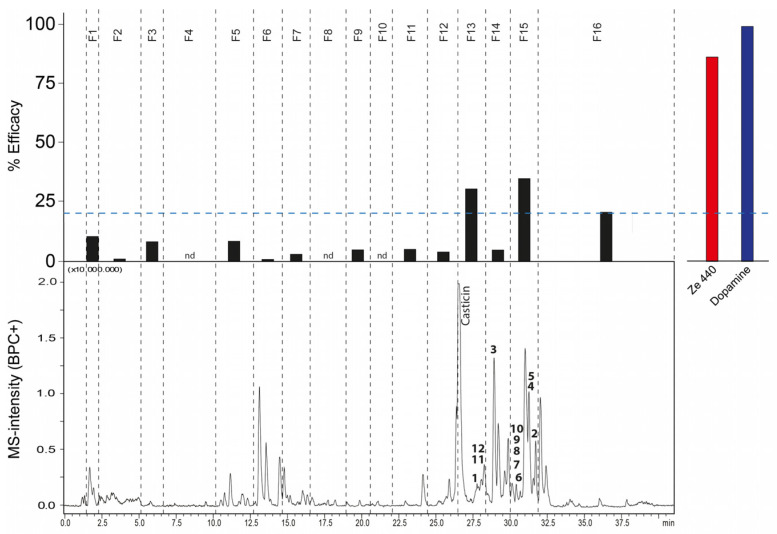
**Ze 440 microfractions show different dopaminergic activities.** Activity of microfractions 1–16 and activity of Ze 440 (60 μg/mL) and dopamine (1 μM) (**top**), together with the ESI-LCMS trace of extract (BPC+; **bottom**). Activity was measured using a functional bioassay in CHO-K1 cells overexpressing the human D2 receptor. Dashed lines indicate the collected microfractions; nd = no activity detected. Numbers in the HPLC chromatogram correspond to isolated compounds **1**–**12**. The dotted line (**top**) shows the efficacy threshold set for selection of microfractions for further analysis.

The VAC extract Ze 440 was then submitted for HPLC-based activity profiling [26,28] to locate compounds with potential dopaminergic activity (Figure 2). Relative to the control (dopamine 1 µM) and the whole VAC extract (60 µg/mL), microfractions 1–12 and 14 exhibited no or only minor activity (top of Figure 2). However, microfractions 13, 15, and 16 exhibited relevant activity and were primarily considered for further compound isolation (top of Figure 2).

### 2.2. Specific Diterpenes and Triterpenes from VAC Extract Activate the D2 Receptor

**Figure 3:**
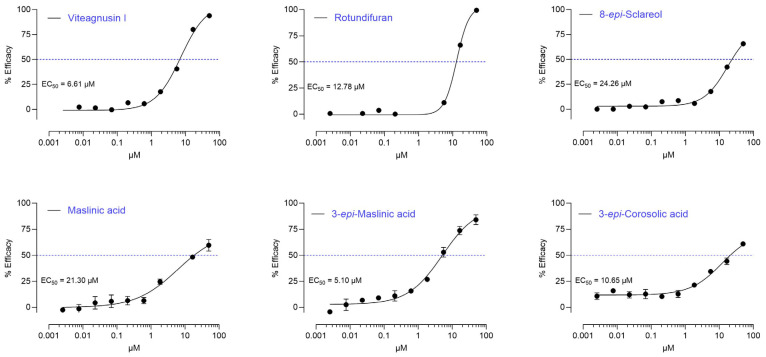
**Specific diterpenes and triterpenes have dopaminergic activity.** Representative EC_50_ profiles at the D2 receptor of identified active compounds (EC_50_ < 50 μM) measured by a functional bioassay in CHO-K1 cells overexpressing the human D2 receptor (n = 2 independent experiments). Dots show the mean of technical triplicates. EC_50_ values were determined using Graphpad Prism 9.0.

**Figure 4:**
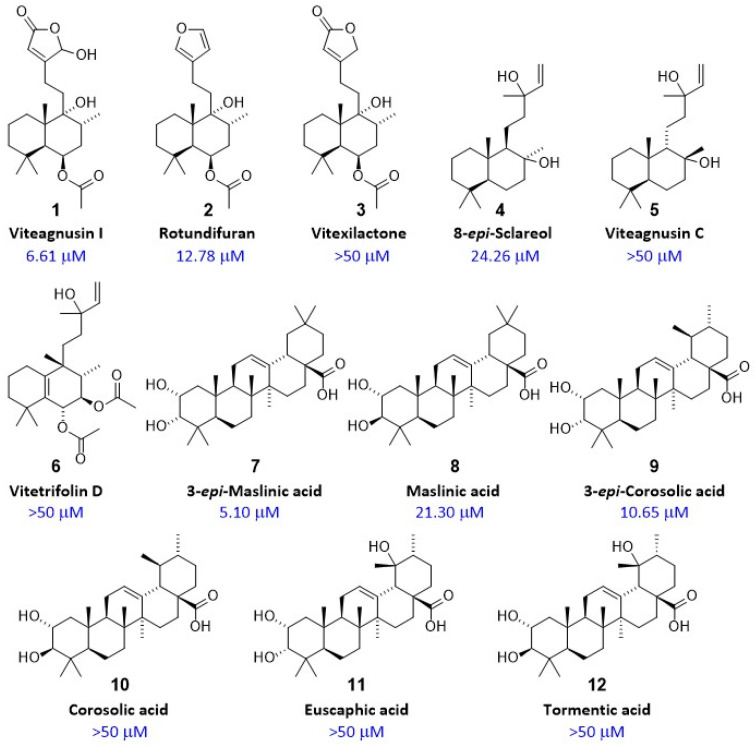
**Overview of chemical structures of isolated diterpenes and triterpenes.** Structures of isolated diterpenes (**1**–**6**) and triterpenes (**7**–**12**), along with their respective EC_50_ for their interaction with the D2 receptor measured using a functional bioassay in CHO-K1 cells overexpressing the human D2 receptor (n = 2 independent experiments). Configurations in the side chains of diterpenes could not be assigned based on NMR data and were thus left open.

Six diterpenes and six triterpenes were isolated from the chromatographic regions corresponding to the two active HPLC microfractions. They were identified by comparing the NMR and ECD data (Supplementary Materials: Tables S1–S4 and Figure S1) with those reported in the literature. The isolated diterpenes and triterpenes were tested using the D2 receptor activity assay (Figure 3), and their chemical structures were compared (Figure 4). Regarding the diterpenes, Viteagnusin I (**1**) from the active microfraction 13 appeared to be the main active diterpene (EC_50_ of 6.6 µM), followed by rotundifuran (**2**; EC_50_ = 12.8 µM, from microfraction 15), whereas vitexilactone (**3**; from the inactive microfraction 14) was inactive (EC_50_ of >50 µM). The diterpenes 3-epi-sclareol (**4**), viteagnusin C (**5**), and vitetrifolin D (**6**) exhibited either negligible or no dopaminergic activity, despite being present in the active microfraction 15.

Interestingly, the pentacyclic triterpenes 3-epi maslinic acid (**7**), maslinic acid (**8**), and 3-epi corosolic acid (**9**) were also found to be active, **7** (EC_50_ of 5.1 µM) being the most active compound (Figure 3 and Figure 4). The triterpenes corosolic acid (**10**), euscaphic acid (**11**), and tormentic acid (**12**), in contrast, were inactive (EC_50_ ≥ 50 µM, Figure 4). Also, the structurally related pentacyclic triterpenes oleanolic acid, arjunolic acid, and ursolic acid, which were not found in the VAC extract, were inactive (EC_50_ ≥ 50 µM). While the biological activity of pentacyclic triterpenoids such as corosolic acid, maslinic acid, and their structural analogues have been reported [40], to our knowledge, this is the first report of D2 receptor activation by pentacyclic triterpenes.

### 2.3. Molecular Modelling Reveals the Binding Modes of Di- and Triterpenes at the D2 Receptor

**Figure 5:**
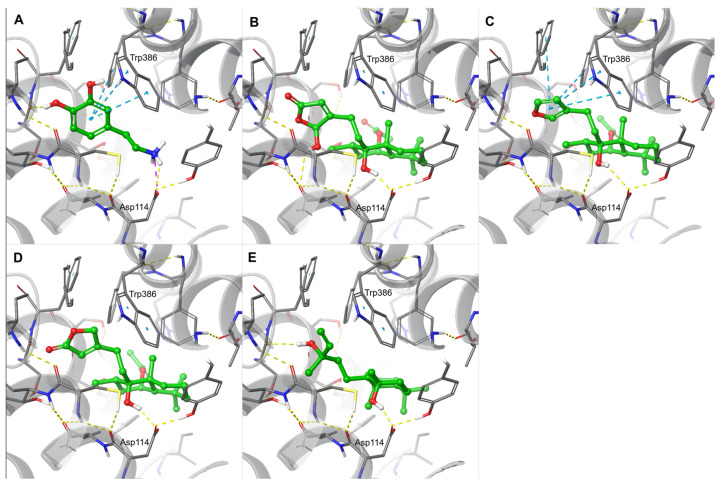
**Schematic illustration of compound binding modes at the D2 receptor predicted via molecular modelling.** Predicted binding modes of (**A**) dopamine, (**B**) viteagnusin I, (**C**) rotundifuran, (**D**) 8-epi-sclareol, and (**E**) vitexilactone docked to the crystal structure with PDB ID 6CM4. Hydrogen bonds are represented by yellow dashed lines, while aquamarine dashed lines show pi–pi interactions. Further color code: blue = nitrogen atoms of ligand; red = oxygen atoms of ligand; green = carbon atoms of ligand; grey = carbon atoms of receptor.

Finally, we used molecular modelling to predict the potential binding modes of the diterpenes and triterpenes at the D2 receptor, thereby facilitating a more comprehensive understanding of the spatial relationships involved (Figure 5). Optimised structures of viteagnusin I (**1**), rotundifuran (**2**), vitexilactone (**3**), 8-epi-sclareol (**4**), viteagnusin C (**5**), vitetrifolin D (**6**), 3-epi-maslinic acid (**7**), and maslinic acid (**8**) were docked at two published crystal structures (PDB IDs 6CM4 and 6LUQ) and an electron microscopy structure (PDB ID 7JVR) of the dopamine D2 receptor. Figure 5 shows predicted binding modes of dopamine and the most active diterpenes **1**–**4** as obtained from molecular docking to the crystal structure with PDB ID 6CM4.

Dopamine, as a reference ligand, is deeply buried within the receptor cavity; the *para*-hydroxy group forms an H-bond with the backbone carbonyl of Ser 197 (referring to 6CM4 residue numbering), and the positively charged primary amine engaging in a salt bridge to Asp 114 (Figure 5A). In the docked pose, the *meta*-hydroxy group is oriented toward the pi-system of Trp 386 but could also form an intramolecular H-bond with the neighbouring hydroxyl (Figure 5A). The phenyl ring of dopamine interacts with nearby aromatic rings (Trp 386 and Phe 390) maintaining the T-shaped geometry (Figure 5A). Thus, dopamine tightly fits the narrow bottom of the binding cavity and displays an ideal, fully saturated pattern of intermolecular interactions.

The most active diterpene **1** fills the binding pocket to a similar degree as dopamine (Figure 5B). The 5-hydroxy group of the 2,5-dihydrofuran-2-one ring donates an H-bond with the carbonyl of Val 115, and the axial hydroxy group on the decaline ring donates an H-bond with Asp 114, at least partially satisfying its H-bonding capacity. Lipophilic carbon atoms fill the pocket with good spatial complementarity, suggesting suitable selectivity towards the D2 receptor (Figure 5B).

Rotundifuran **2** and Vitexilactone **3,** as close structural analogues of Viteagnusin I **1**, despite being docked to the same pocket and providing the interaction to Asp 114, are not able to form H-bonds from the five-membered rings to the protein (Figure 5C,E). This leads to a significant decrease in the biological activity because of uncompensated desolvation costs of buried polar atoms. In the case of Rotundifuran **2**, the loss is partially compensated by multiple T-shaped interactions with nearby aromatic sidechains, making it the second most active diterpene in our set (Figure 5C). Interestingly, 8-*epi*-Sclareol **4** is able to form an H-bond with the carbonyl of Ser 197 (Figure 5D). However, due to increased flexibility, such an H-bond may not be as stable as for analogues featuring five-membered heterocycles, and the molecule also suffers from a larger entropic penalty upon binding.

Finally, the doubly acetylated diterpene vitetrifolin **6** was found to be too bulky to be able to fit into the binding pocket.

Unfortunately, molecular docking of the rather bulky and rigid triterpenes **7**–**12** did not yield any poses that would allow 3D mechanistic insights at the atomic level.

## 3. Discussion

The first report on the dopaminergic activity of VAC extracts and fractions thereof were published in the 1990s [20]. In this study, the fractionation of a water-soluble, commercially available extract was performed by pressure filtration through ‘molecular sieves’ with further separation steps using Sephadex LH20. The resulting fractions exerted very weak dopaminergic activity. In a follow-up study by Berger [18], VAC fruit extract and a hexane fraction thereof showed a weak D2 receptor affinity with IC_50_ values of 52 µg/mL and 32 µg/mL, respectively. In all of the previous pharmacological studies, dopaminergic activity was determined either using D2 receptor radioligand binding assays or indirectly by measuring the decrease in PRL in anterior pituitary cell culture supernatants. In two of these studies, the suppression of forskolin-induced cAMP production was measured in rat anterior pituitary cells after treatment with so-called ‘BNO-diterpenes’ or ‘clerodienols’ [17,19]. However, it remains unclear which of the VAC diterpenes were specifically tested. Furthermore, no data on their efficacy and potency were reported [26,28].

In the present study, this topic was therefore re-examined using an HPLC activity profiling-based approach [26,28]. The D2 receptor bioassay was used as a tool to track dopaminergic activity in the VAC extract Ze 440 as well as in the obtained HPLC microfractions. Upon binding of an agonist to the D2 receptor, the Gi/o protein is activated, leading to a decrease in cAMP signal via adenylate cyclase inhibition. The VAC extract Ze 440, using VAC fruits of the Vitan variety only [39], was found to potently activate the D2 receptor. Accordingly, HPLC-based activity profiling [26,28] for locating compounds with potential dopaminergic activity resulted in specific fractions that exhibited relevant activity and were considered for further compound isolation.

We isolated the diterpenes viteagnusin I (**1**) [41,42], rotundifuran (**2**) [43], vitexilactone (**3**) [41], 8-*epi*-sclareol(**4**) [44,45], viteagnusin C (**5**) [44], and vitetrifolin D (**6**) [44,46] as well as the pentacyclic triterpenes 3-*epi*-maslinic acid (**7**) [47], maslinic acid (**8**) [48], 3-*epi*-corosolic acid (**9**) [47], corosolic acid (**10**) [49], euscaphic acid (**11**) [50], and tormentic acid (**12**) [51]. The occurrence of diterpenes **1**–**6** in fruits of VAC has been described previously [41,42,44,49], and the dopaminergic activity of some of these diterpenes has been reported [17,18,19,20,39]. While **7**–**10** have been previously found in the fruits of VAC [49], **11** and **12** have, so far, only been reported from other *Vitex* species [52]. Functional and structural analyses of these six diterpenes (**1**–**6**) and six triterpenes (**7**–**12**) revealed that the dopaminergic activity of VAC diterpenes seems to depend on the configuration and on ring substitution in the side chain. In the series of structures of **1**, **2**, and **3**, the most active (**1**) contains a hydroxy-substituted furanone ring, while no activity was found in the absence of a hydroxy group as in **3**. In comparison, the furan-containing compound **2** was still considerably active but less so than **1**. This strongly hints at the influence of the hydrogen bond donor/acceptor pattern on the interaction with the D2 receptor. The other notable difference was found between **4** and **5**, which only differ in their configurations at C-8 and C-9. This alters the three-dimensional structure sufficiently to cause a loss of observed activity. Regarding the activity of triterpenes, it has been reported before that pentacyclic triterpenoids such as corosolic acid, maslinic acid, and their structural analogues exert biological activity [40]. However, to our knowledge, this is the first report of D2 receptor activation by pentacyclic triterpenes. Interestingly, the observed activity on the D2 receptor changes 4-fold between the epimers **7** and **8** and >5-fold between epimers **9** and **10**, with both pairs differing only in their configuration at C3. A 2-fold decrease in activity is observed between **7** and **9**, which differ only in the positioning of CH_3_-29 on the E-ring. This, together with the lack of activity in related pentacyclic triterpenes, hints at a specific interaction of 3-*epi*-maslinic acid (**7**) and close derivatives with the D2 receptor.

Molecular modelling is a strong tool for predicting the binding of potential ligands to a specific receptor. Our molecular modelling approach revealed that, in contrast to dopamine, none of the studied isoprenoids featured a positively charged (amine) group that could interact with Asp 114 to form a salt bridge. This is probably the main reason for the generally weaker binding affinities observed with most compounds when compared to basic amines like dopamine or other known D2 receptor ligands.

The poor activity of diterpene **5**, a stereoisomer of **4**, in the D2 receptor bioassay clearly demonstrates the importance of the H-bond donated to the key binding site residue Asp 114. None of the docked poses of **5** could occupy the bottom part of the binding cavity and simultaneously donate an H-bond with Asp 114.

Compared to the neutral diterpenes, all the studied triterpenes feature a carboxylic group. This further complicates finding reasonable binding modes upon docking, given that the dopamine D2 receptor ligand binding cavity has no positively charged counterpart residues (none within a 5Å distance from the cocrystallised ligand as seen in PDB ID: 6CM4). In addition to that, the negatively charged residue Asp 114 at the very heart of the binding pocket creates a major obstacle for docking due to electrostatic repulsion towards the triterpene carboxyl group.

Nevertheless, some elementary structure–activity relationships and binding preferences can be assumed by looking at the structural formulae of the triterpenes alone. The fact that the biological activity of triterpenes depends on the spatial configuration of adjacent hydroxyls would suggest that triterpenes could engage in interactions with serine and threonine residues (4× and 2× within a 5Å radius from the ligand in PDB ID: 6CM4, respectively) involved in a cooperative H-bond network in the binding pocket (e.g., Ser 197, Thr 119, and Ser 193). Such residues are lining the narrower, buried part of the ligand pocket and could be addressed if induced-fit effects were realistically considered upon docking. We tried to cope with this problem by including an ensemble of various receptor conformations as they naturally occur in crystal or cryoEM structures. However, docking programs in our study assume rigidity of the protein structure, meaning that due to steric hindrance and electronic repulsion, certain parts of the binding cavity may remain “out of reach” for bulky, rigid, and negatively charged ligands like triterpenes.

In conclusion, six diterpenes were isolated from active HPLC microfractions 13 and 15. Viteagnusin I (**1**) was identified as the most active diterpene (IC_50_ of 6.6 µM), followed by rotundifuran (**2**; EC_50_ of 12.8 µM). Vitexilactone (**3**) was inactive (EC_50_ > 50 µM). The diterpenes 8-*epi*-sclareol (**4**), vitetrifolin D (**5**), and viteagnusin C (**6**) showed either weak or no dopaminergic activity. Interestingly, triterpenes were also identified as active, with 3-*epi*-maslinic acid (**7**) being the most active compound (EC_50_ of 5.1 µM). The findings from the docking study of selected diterpenes corroborated the in vitro data. The observed differences in H-bonding patterns and steric accessibility based on the predicted binding modes of diterpenes could be correlated with the biological activity. However, the docking of bulky, rigid triterpene ligands did not lead to any conclusive results and remains challenging due to uncertainty about induced-fit effects and electrostatic repulsion.

Taken together, our data show for the first time that the dopaminergic activity of VAC diterpenes seems to depend on the configuration and on ring substitution in the side chain. Furthermore, we demonstrate for the first time that triterpenes such as 3-*epi*-maslinic acid (**7**) contribute to the overall dopaminergic activity of VAC extract Ze 440. Numerous VAC-containing pharmaceutical products and food supplements are commercially available worldwide. However, the findings reported here cannot be extrapolated to VAC extracts other than Ze 440 due to the use of a proprietary chaste tree variety and extraction procedure, which leads to a unique phytochemical profile of the extract [39].

These findings contribute to a more comprehensive understanding of the mechanism of action of VAC extracts, for example, in the context of PMS symptoms.

## 4. Materials and Methods

Chemicals, reagents, and plant extract. Dried *Vitex agnus castus* fruits (3 kg; batch 200510) were ground (particle size < 1 mm) and extracted with 60% (*m*/*m*) ethanol at 45–50 °C. After sedimentation at room temperature for 1 h, the ethanol was evaporated to yield a spissum extract with a dry weight of 74.14%. The drug extract ratio (DER) of the obtained spissum extract was within the specification of 6–12:1. Analytical and semi-preparative separations were performedusing HPLC-MS-grade solvents (acetonitrile and formic acid; Macron Fine Chemicals, Radnor, PA, USA) and ultrapure water from a Milli-Q water purification system (Merck Millipore, Billerica, MA, USA). For extraction, liquid–liquid partitioning, and preparative separations technical grade solvents were used after distillation. Reference compounds vitexilactone (purity > 97%, BOC Sciences, Shirley, NY, USA), casticin (purity > 98%, Sigma-Aldrich, St. Louis, MO, USA), ursolic acid (purity > 90%, Sigma-Aldrich), and oleanolic acid (purity > 95%, Roth, Karlsruhe, Germany) were purchased, and arjunolic acid (purity > 95%) was previously isolated from *Miconia affinis* [53].

### 4.1. HPLC-Based Activity Profiling

HPLC-based activity profiling was performed according to established methodologies [27,28]. In short, the spissum extract was dried in vacuo (<0.5 mbar), and 15.6 mg of dry mass was dissolved in DMSO at a concentration of 50 mg/mL, sonicated, and centrifuged at 18× *g* for 7 min. The supernatant was separated utilizing a 1290 Infinity II Preparative LC system consisting of a binary pump (1260 Prep Bin pump) connected to a 1100 Series photodiode array detector (PDA) (all equipment: Agilent Technologies, Santa Clara, CA, USA). The extract was injected onto a semi-preparative HPLC column (SunFire C_18_, 5.0 μm, 150 × 10.0 mm i.d., Waters, Milford, MA, USA) in three portions of 5 mg each using gradient elution [solvent A: water B: acetonitrile; 0–2 min 5% B, 2–22 min 5 → 45% B, 22–30 min 45 → 100% B, 30–40 min 100% B; flow rate 4 mL/min; injection volume 100 μL]. A total of 16 fractions were collected between 1 min and 40 min and dried in vacuo. One-third of each collected fraction was used for HLPC-PDA-CAD-MS analysis, while two-thirds were used to test for dopaminergic activity. The insoluble residue after centrifugation of the extract was collected, dried, and tested for dopaminergic activity as a separate fraction. HLPC-PDA-CAD-MS analysis on an LC-MS 8030 system (Shimadzu, Kyoto, Japan)—consisting of a degasser, binary high-pressure mixing pump, autosampler, column oven (CTO-20AC), and PDA detector (SPD-M20A)—was carried out on a Waters SunFire C_18_ column [3.5 μm, 150 × 3.0 mm i.d.; solvent A: water + 0.1% formic acid (FA), B: acetonitrile + 0.1% FA; 0–2 min 5% B, 2–22 min 5 → 45% B, 22–30 min 45 → 100% B, 30–40 min 100% B; flow rate 0.5 mL/min]. A Corona Veo RS Charged Aerosol Detector (Thermo Fisher Scientific, Waltham, MA, USA) was connected via a T-splitter between the PDA detector and the triple-quadrupole MS. Retention times for all isolated compounds were recorded with an optimised gradient on a Waters SunFire C_18_ column [3.5 μm, 150 × 3.0 mm i.d.; solvent A: water + 0.1% formic acid (FA), B: acetonitrile + 0.1% FA; 0–2 min 60% B, 2–22 min 60 → 80% B, 22–30 min 80 → 100% B, 30–36 min 100% B; flow rate 0.5 mL/min].

### 4.2. Isolation and Identification of Compounds

The ethanolic spissum extract (266 g) was extracted three times with 500 mL of EtOAc via liquid–liquid extraction. The organic layers were collected and dried over Na_2_SO_4_, and the solvent was removed in vacuo to yield 41 g of an EtOAc fraction. An aliquot of this fraction (24 g) was subjected to MPLC on a silica gel column (40–63 μm, 40 × 7.5 cm i.d.) using a gradient of 5 → 50% EtOAc in hexane over 3 h at a flow rate of 20 mL/min. This was followed by stepwise increments to 100% EtOAc and a wash with 100% MeOH. A total of 23 fractions (A-W) were collected. The separation of fraction F on a Waters SunFire C_18_ column (5 μm, 150 × 40 mm i.d.; 20 mL/min) with a gradient [solvent A: H_2_O + 0.1% FA, B: acetonitrile + 0.1% FA; 0–3 min 60% B, 3–23 min 60 → 80% B; injection: 76.9 mg dissolved in 325 uL DMSO + 325 μL MeCN/H_2_O 2:1] yielded 8.2 mg of vitetrifolin D (**6**), t_R_ 13.0 min.

The separation of fraction G (1.76 g dissolved in 3 mL of MeOH) on a Sephadex LH-20 column (100 × 2.5 cm i.d.) eluted with MeOH at a flow rate 1.5 mL/min afforded 11 fractions. Recrystallisation of fraction 8 yielded 5-hydroxy-3,4′,6,7-tetramethoxyflavone (33.7 mg, t_R_ 7.6 min). Fraction 3 (424.7 mg) was separated on a SunFire C_18_ column using gradient elution (solvent A: H_2_O + 0.1% FA, B: acetonitrile + 0.1% FA; 0–3 min 80% B, 3–23 min 80 → 90% B, 23–24 min 90 → 100%; injection: 50–100 mg in MeOH) to afford a mixture, which was submitted to semi-preparative HPLC (SunFire C_18_, 5.0 μm, 150 × 10 mm i.d.) using a gradient of acetonitrile (B) in H_2_O (A) containing 0.1% FA [0–3 min 62% B, 3–23 min 62 → 67% B, 23–24 min 67 → 100%; flow rate 5 mL/min; injection: 2–20 mg in MeOH]. Viteagnusin C (**5**, 5.5 mg, t_R_ 12.8 min) and 8-epi-sclareol (**4**, 16.2 mg, t_R_ 14.1 min) were obtained.

Fraction D was separated into 2 portions (60 mg each in 300 μL of acetonitrile) on a SunFire C_18_ column eluted with 80% B to afford rotundifuran (**2**, 75 mg, t_R_ 17.4 min).

Time-based separation of fraction J (262 mg) on a Sephadex LH-20 column (100 × 1 cm i.d.; MeOH; flow rate 0.6 mL/min) gave 57 fractions of 10 min each. The dry residue of fractions 19–24 (56 mg) was separated on a SunFire C_18_ column (5.0 μm, 150 × 10 mm i.d.) using a gradient [0–2 min 50% B, 2–13 min 50 → 54% B, 13–23 min 54%; 6 injections of 2–11 mg each in MeOH] to yield viteagnusin I (**1**, 6.2 mg, t_R_ 27.8 min) and vitexilactone (**3**, 9.8 mg, t_R_ 6.9 min).

Fraction M (637 mg) was recrystallised several times from MeOH to remove casticin (297 mg, t_R_ 3.8 min), and 22.2 mg of a residue rich in triterpenes was obtained. This residue was separated on a ReproSil-Pur 120 C18-AQ column (3.0 μm, 150 × 10 mm i.d.; Dr. Maisch, Ammerbuch, Germany) eluted at a flow rate of 5 mL/min with H_2_O/MeOH (22:78) containing 0.1% FA. Maslinic acid (**8**, 2.9 mg, t_R_ 10.7 min), corosolic acid (**10**, 4.0 mg, t_R_ 11.2 min), and 1.7 mg of a mixture of two triterpenes were obtained. The mixture was re-injected on the same column eluted with 66% MeOH to afford euscaphic acid (**11**, 0.3 mg, t_R_ 5.0 min) and tormentic acid (**12**, 0.7 mg, t_R_ 5.0 min).

Fractions O (391 mg) and K (400 mg) were each dissolved in MeOH and centrifuged, and the supernatants were injected onto a Sephadex LH-20 column (100 × 1.5 cm i.d.; MeOH; flow rate 0.5 mL/min). The triterpene-containing fractions were combined and dried to yield two subfractions of 51 mg each. The separation of the triterpene fraction from O on a ReproSil-Pur 120 C18-AQ column (3.0 μm, 150 × 10 mm i.d.) eluted with H_2_O/MeOH (22:78) containing 0.1% FA yielded additional maslinic acid (**8**, 0.6 mg, t_R_ 10.7 min), corosolic acid (**10**, 1.3 mg, t_R_ 11.2 min), and 7 mg of a triterpene mixture. The mixture was separated with 66% MeOH to afford additional tormentic acid (**12**, 4.8 mg, t_R_ 5.0 min). The triterpene-containing fraction of K was separated on the same column eluted with 76% MeOH to obtain 3-*epi*-maslinic acid (**7**, 2.5 mg, t_R_ 10.2 min) and 3-*epi*-corosolic acid (**9**, 2.2 mg, t_R_ 10.7 min).

ECD and UV spectra were recorded in MeOH (83.25–333.3 μg/mL) on a Chirascan CD spectrometer (Leatherhead, UK) using 1 mm path precision cells (110 QS, Hellma Analytics, Müllheim, Germany). NMR spectra were recorded on an Avance III NMR spectrometer operating at 500.13 MHz for ^1^H and 125.77 MHz for ^13^C, equipped with a 1 mm TXI microprobe or a 5 mm BBO probe (all equipment: Bruker, Fällanden, Switzerland). The spectra were recorded at 23 °C in CD_3_OD (Armar Chemicals, Döttingen, Switzerland) and analysed by Bruker TopSpin 3.5 and ACD/Labs NMR Workbook suite software version 2022.2.3 (Toronto, ON, Canada). Chemical shifts are reported as δ values (ppm) and residual signals as internal reference, J in Hz.

Viteagnusin I (**1**), ${\left[\alpha \right]}_{D}^{25}$ −2.3 (c 0.9 mg/mL, MeOH); ECD (c 0.3 mg/mL, MeOH) 207 (∆ε + 1.8); for ^1^H and ^13^C NMR data, see Tables S1 and S2, Supplementary Materials.

Rotundifuran (**2**), ${\left[\alpha \right]}_{D}^{25}$ −14.1 (c 1.0 mg/mL, MeOH); ECD (c 0.3 mg/mL, MeOH) 211 (∆ε + 1.8); for ^1^H and ^13^C NMR data see Tables S1 and S2, Supplementary Materials.

Vitexilactone (**3**), ${\left[\alpha \right]}_{D}^{25}$ −5.3 (c 1.1 mg/mL, MeOH); ECD (c 0.3 mg/mL, MeOH) 213 (∆ε + 2.1).

8-*epi*-Sclareol (**4**), ${\left[\alpha \right]}_{D}^{25}$ −7.9 (c 1.3 mg/mL, MeOH); ECD (c 0.3 mg/mL, MeOH) 195 (∆ε + 1.0); for ^1^H and ^13^C NMR data, see Tables S1 and S2, Supplementary Materials.

Viteagnusin C (**5**), ${\left[\alpha \right]}_{D}^{25}$ −0.6 (c 0.7 mg/mL, MeOH); ECD (c 0.3 mg/mL, MeOH) 195 (∆ε + 0.8); for ^1^H and ^13^C NMR data, see Tables S1 and S2, Supplementary Materials.

Vitetrifolin D (**6**), ${\left[\alpha \right]}_{D}^{25}$ 126 (c 0.8 mg/mL, MeOH); ECD (c 0.3 mg/mL, MeOH) 198 (∆ε − 5.0), 215 (∆ε − 12.2); for ^1^H and ^13^C NMR data, see Tables S1 and S2, Supplementary Materials.

3-*epi*-Maslinic acid (**7**); for ^1^H and ^13^C NMR data, see Tables S3 and S4, Supplementary Materials.

Maslinic acid (**8**); for ^1^H and ^13^C NMR data, see Tables S3 and S4, Supplementary Materials.

3-*epi*-Corosolic acid (**9**); for ^1^H and ^13^C NMR data, see Tables S3 and S4, Supplementary Materials.

Corosolic acid (**10**); for ^1^H and ^13^C NMR data, see Tables S3 and S4, Supplementary Materials.

Euscaphic acid (**11**); for ^1^H and ^13^C NMR data, see Tables S3 and S4, Supplementary Materials.

Tormentic acid (**12**); for ^1^H and ^13^C NMR data, see Tables S3 and S4, Supplementary Materials.

### 4.3. Dopamine Assay

Dopaminergic activity was determined with a functional assay in CHO-K1 cells overexpressing the human D2 receptor, using a cAMP read-out (Eurofins DiscoverX Corporation, Fremont, CA, USA). The cells (5000 cells/cm^2^) were seeded in a total volume of 20 μL onto white 384-well microplates and incubated at 37 °C for 18 h prior to testing. cAMP modulation was determined using the DiscoverX HitHunter cAMP XS+ assay. Since the D2 receptor couples mainly to G_i/o_ (inhibitory towards cAMP), the assay was run in G_i/o_ agonist format. In brief, the cells were incubated with the samples in the presence of 25 µM forskolin to induce a response. Medium was aspirated from cells and replaced with 10 µL HBSS/10 mM Hepes (assay buffer). Intermediate dilution of sample stocks was performed in the assay buffer to generate a concentration 4-fold higher (4×) than the final in-well concentration. A total of 5 µL of 4× sample was added to the cells, followed by 5 µL 4× EC80 forskolin in the assay buffer and incubated at 37 °C for 30 min. The final DMSO concentration (vehicle) was 1%. After appropriate compound incubation, the assay signal was generated through incubation with 5 µL of cAMP XS + Ab reagent followed by 20 μL cAMP XS + ED/CL lysis cocktail for one hour followed by incubation with 20 μL cAMP XS + EA reagent for three hours at room temperature. The microplates were read following signal generation with an Envision^TM^ instrument (PerkinElmer, Waltham, MA, USA) for chemiluminescent signal detection. A control dose curve analysis was performed using dopamine as agonist. The data shown were normalised to the maximal (EC_100_ dopamine) and minimal (vehicle) responses.

### 4.4. Molecular Modelling

Two crystal structures (PDB IDs 6CM4 and 6LUQ) and one electron microscopy structure (PDB ID 7JVR) of the dopamine D2 receptor were computationally prepared using Schrödinger’s Maestro version 2021-1 [54]. Their preparation consisted of the addition of explicit hydrogen atoms, the assignment of correct bond orders, the addition of missing side chains and loops, as well as the generation of correct protonation states at physiological pH [55,56,57,58]. Since the two crystal structures contained engineered mutations, they were adjusted to the wild type. This involved the mutations A122I, A375L, and A379L for 6CM4, and A122I, A396L, and A400L for 6LUQ. To complete the preparation, the H-bonding network was optimised using Maestro’s Protein Preparation Wizard, and a restrained minimisation was run using the OPLS 2005 force field and a 0.3 Å convergence threshold for heavy atoms.

Structures of dopamine, viteagnusin I (**1**), rotundifuran (**2**), vitexilactone (**3**), 3-epi-sclareol (**4**), viteagnusin C (**5**), vitetrifolin D (**5**), 3-epi-maslinic acid (**7**), and maslinic acid (**8**) were prepared using Schrödinger’s LigPrep using the OPLS4 force field, and protonation states at pH 7.4 were generated [59]. All compounds were then docked to the prepared protein structures using smina [60], Glide [61], and LeDock [62]. For the compounds with ambiguous stereochemistry, we docked and analysed all possible stereoisomers. All top-ranked poses generated by docking programs were subjected to visual analysis according to the quality criteria published by Fischer et al. [63].

### 4.5. Data Analysis

For Gi agonist mode assays, percentage activity was calculated using the following formula: % Activity = 100% × (1 − (mean RLU of test sample − mean RLU of MAX control)/(mean RLU of vehicle control − mean RLU of MAX control)). Concentration–response curves were generated using EC_50_ using Graphpad Prism version 9.0 (Graphpad Software, Boston, MA, USA), and curve fitting was performed using non-linear regression analysis applying the following variable slope equation: Y = Bottom + (Top − Bottom)/(1 + 10^((LogEC_50_ − X) × HillSlope)).

## Data Availability

The data presented in this study are available on request from the corresponding authors.

## Supplementary Materials

The following supporting information can be downloaded at https://www.mdpi.com/article/10.3390/ijms252111456/s1.
