# Supplementary material for: Vitex agnus castus Extract Ze 440: Diterpene and Triterpene’s Interactions with Dopamine D2 Receptor
Source: Int J Mol Sci. 2024 Oct 25;25(21):11456. doi: 10.3390/ijms252111456 (PMC11547015; doi:10.3390/ijms252111456)
Supplement: Supplementary file 1 [file ijms-25-11456-s001.zip › ijms-3254766-supplementary.pdf]

Supplementary Materials

Table S1. ^{13}C NMR spectroscopic data for compounds **1-6** (MeOD; 126 MHz; δ in ppm).

Atom	Viteagnusin I (**1**)	Rotundifuran (**2**)	Vitexilactone (**3**)	8-epi-Sclareol (**4**)	Viteagnusin C (**5**)	Vitetrifolin D (**6**)
**1**	34.8	33.5	34.8	40.8	37.9	25.9
**2**	19.9	18.6	19.9	19.5	19.8	19.4
**3**	45	43.6	45.0	43.5	43.6	39.3
**4**	35	33.8	35.1	34.2	34.0	34.6
**5**	48.7	47.4	48.7	57.7	47.9	132.5
**6**	71.9	70.2	71.9	19.7	21.7	66.2
**7**	37.3	36.0	37.3	43.3	38.3	72.7
**8**	33	31.7	33.0	74.2	74.5	36.4
**9**	77.8	76.9	77.8	61	62.8	42.9
**10**	45.3	43.6	45.3	40.6	40.2	141.5
**11**	32.6	34.7	33.0	20.7	22.8	29.3
**12**	25.8	21.4	26.5	47.8	47.0	38.6
**13**	173.4	125.4	175.5	74.5	74.7	73.1
**14**	117.3	110.7	115.0	146.4	146.7	144.4
**15**	173.9	142.9	177.3	112.4	112.0	112.1
**16**	101.2	138.4	75.2	27.4	27.8	112.1
**17**	16.6	16.0	16.6	31.2	32.2	28.1
**18**	34.2	33.5	34.2	34.3	33.8	28.1
**19**	24.4	23.6	24.4	22.4	21.9	29.3
**20**	20.1	18.9	20.1	15.9	25.6	11.1
**1'**	172.7	170.6	172.6			170.8
**2'**	22.0	21.9	22.0			21.4
**1''**						170.8
**2''**						20.9

Table S2. ^{1}H-NMR spectroscopic data for compounds **1-6** (MeOD; 500 MHz; δ in ppm, coupling constants in Hz).

**Atom**	Viteagnusin I (**1**)	Rotundifuran (**2**)	Vitexilactone (**3**)	8-epi-Sclareol (**4**)	Viteagnusin C (**5**)	Vitetrifolin D (**6**)
**1a**	1.68, m	1.48, m	1.68, m	1.71, m	1.09, m	2.02, br dd (7.0, 5.5)
**1b**	1.48, m		1.47, m		1.68, m	
**2a**	1.49, m	1.49, m	1.50, m	1.4, m	1.42, m	1.62, m
**2b**	1.69, m	1.65, m	1.68, m	1.64, m	1.71, m	
**3a**	1.21, m	1.16, m (13.2, 13.2, 3.2)	1.22, m	1.17, td (13.5, 13.5, 4.1)	1.2, dd (13.1, 3.4)	1.48, dd (8.7, 3.8)
**3b**	1.34, m	1.32, m	1.34, m	1.38, m	1.37, m	
**5**	1.74, d (2.1)	1.65, d (1.8)	1.74, d (2.1)	0.86, m	1.12, br dd (12.7, 2.9)	
**6a**	5.38, dt (2.6, 2x2.6)	5.38, dt (3.0, 2x2.4)	5.38, dt (2.7, 2x2.7)	1.47, m	1.31, m	5.6, d (3.3)
**6b**				1.6, m	1.56, m	
**7a**	1.47, m	1.64, ddd (14.2, 13.4, 3.1)	1.48, m	1.42, m	1.51, m	4.85, dd (13.0, 3.3)
**7b**	1.69, m	1.53, ddd (14.2, 3.4, 3.4)	1.69, m	1.74, m		
**8**	2.15, m	2.11, m	2.17, dqd (13.0, 3x6.7, 4.0)			2.06, dd (13.0, 7.3)
**9**				0.7, dd (3.4, 3.4)	0.95, t (2.9, 2.9)	
**11a**	1.95, m	1.76, ddd (14.5, 10.5, 5.8)	1.83, ddd (14.4, 10.8, 6.0)	1.34, m	1.42, m	1.42, m
**11b**	1.82, m	1.91, ddd (14.5, 11.1, 6.4)	1.95, ddd (14.4, 11.2, 6.3)	1.44, m	1.79, m	
**12a**	2.48, m	2.51, m	2.58, m	1.57, m	1.49, m	1.5, m
**12b**	2.59, m				1.59, m	1.18, m
**14**	5.89, m	6.28, br s	5.88, dddd (1.5, 1.5, 1.5, 1.5)	5.93, dd (17.4, 11.0)	5.93, dd (17.4, 10.7)	5.83, dd (17.3, 10.8)
**15a**		7.35, dd (1.5, 1.5)		5.04, dd (11.0, 1.5)	5.2, dd (17.4, 1.5)	5.17, dd (17.3, 1.2)
**15b**				5.2, dd (17.4, 1.5)	5.01, dd (11.0, 1.5)	5.05, dd (10.8, 1.1)
**16**	6.02, s	7.22, br s	4.87, d (1.5)	1.26, s	1.25, s	1.25, s
**17**	0.92, d (6.7)	0.93, d (6.7)	0.91, d (6.7)	1.12, s	1.43, s	1.06, s
**18**	0.95, s	0.95, s	0.95, s	0.87, s	0.87, s	0.9, s
**19**	1.03, s	0.99, s	1.03, s	0.85, s	0.81, s	1.06, s
**20**	1.31, s	1.25, s	1.30, s	0.97, s	1.1, s	0.9, d (7.0)
**2'**	2.04, s	2.04, s	2.03, s			2.03, s
**2''**						1.98, s
**8-OH**					4.61, br s	
**13-OH**					4.61, br s	

Table S3. ^{13}C NMR spectroscopic data for compounds **7-12** (MeOD; 126 MHz; δ in ppm).

**Atom #**	3-epi-Maslinic acid (**7**)	Maslinic acid (**8**)	3-epi-Corosolic acid (**9**)	Corosolic acid (**10**)	Euscaphic acid (**11**)	Tormentic acid (**12**)
**1**	42.6	48.3	42.8	48.4	42.6	48.3
**2**	67.3	69.6	67.3	69.7	67.3	69.7
**3**	80.2	84.6	80.2	84.6	80.3	84.7
**4**	39.6	40.7	39.6	40.7	39.5	40.7
**5**	49.4	56.8	49.4	56.8	49.4	56.8
**6**	19.3	19.7	19.3	19.7	19.4	19.8
**7**	34.0	34.1	34.3	34.4	34.2	34.2
**8**	40.9	40.7	41.1	41.0	41.4	41.2
**9**	48.9	49.2	48.7	49.1	48.3	48.8
**10**	39.6	39.4	39.5	39.3	39.5	39.3
**11**	24.7	24.8	24.5	24.6	24.9	24.9
**12**	123.7	123.6	126.9	126.8	129.5	129.4
**13**	145.5	145.6	139.9	140.0	140.2	140.2
**14**	43.2	43.1	43.5	43.5	42.9	42.8
**15**	28.9	29.0	29.3	29.3	29.7	29.7
**16**	24.2	24.2	25.5	25.5	26.7	26.7
**17**	47.8	47.8	49.3	49.3	49.1	49.3
**18**	42.9	42.9	54.5	54.5	55.2	55.2
**19**	47.4	47.4	40.6	40.6	73.7	73.7
**20**	31.8	31.8	40.6	40.6	43.2	43.2
**21**	35.0	35.1	31.9	31.9	27.4	27.4
**22**	34.0	34.0	38.3	38.3	39.2	39.2
**23**	29.4	29.4	29.4	29.5	29.4	29.5
**24**	22.6	17.6	22.6	17.7	22.6	17.6
**25**	17.0	17.2	17.2	17.4	17.0	17.2
**26**	17.9	17.9	18.0	18.0	17.7	17.6
**27**	26.7	26.6	24.3	24.2	25.1	25.0
**28**	182.1	172.8	181.9	182.0	182.5	182.4
**29**	33.7	33.7	17.8	17.8	27.2	27.2
**30**	24.1	24.1	21.7	21.7	16.8	16.8

Table S4. ^{1}H-NMR spectroscopic data for compounds **7-12** (MeOD; 500 MHz; δ in ppm, coupling constants in Hz).

Atom #	3-epi-Maslinic acid (**7**)	Maslinic acid (**8**)	3-epi-Corosolic acid (**9**)	Corosolic acid (**10**)	Euscaphic acid (**11**)	Tormentic acid (**12**)
**1a**	1.26, m	0.91, m	1.59, br dd (12.1, 4.1)	1.96, m	1.57, m	1.94, dd (12.5, 4.0)
**1b**	1.56, m	1.93, m	1.27, m	0.9, m	1.3, m	0.91, m
**2**	3.93, ddd (11.9, 4.0, 3.1)	3.62, ddd (11.3, 9.7, 4.6)	3.93, ddd (11.9, 4.3, 3.1)	3.63, ddd (11.0, 9.5, 4.6)	3.93, ddd (11.9, 4.3, 2.7)	3.63, ddd (11.4, 9.7, 4.0)
**3**	3.32, m	2.91, d (9.7)	3.32, m	2.91, d (9.5)	3.33, d (2.7)	2.92, br d (9.7)
**5**	1.24, m	0.85, m	1.24, m	0.85, m	1.27, m	0.87, m
**6a**	1.47, m	1.57, m	1.46, m	1.56, m	1.39, m	1.44, m
**6b**	1.4, m	1.44, m	1.39, m	1.42, m	1.47, m	1.56, m
**7a**	1.74, m	1.33, m	1.58, m	1.56, m	1.6, m	1.33, m
**7b**	1.31, m	1.75, m	1.33, m	1.36, m	1.31, m	1.58, m
**9**	1.76, m	1.65, m	1.73, m	1.6, m	1.87, m	1.75, m
**11**	1.94, m	1.94, m	1.97, m	1.97, m	2.01, m	2.01, m
**12**	5.26, t (2x3.4)	5.25, t (2x3.2)	5.24, t (2x3.5)	5.24, t (2x3.5)	5.3, t (2x3.4)	5.29, br t (2x3.4)
**15a**	1.79, m	1.07, br ddd (13.7, 3.4, 3.4)	1.09, m	1.93, m	1, m	0.99, m
**15b**	1.08, ddd (13.6, 3.4, 3.4)	1.8, dd (13.7, 4.0)	1.94, m	1.08, m	1.81, m	1.81, td (13.7, 13.1, 4.0)
**16a**	1.6, m	2.02, m	2.05, ddd (13.7, 13.7, 4.0)	1.65, m	1.51, m	1.52, m
**16b**	2.02, ddd (13.6, 13.6, 4.0)	1.6, m	1.65, m	2.03, ddd (13.4, 13.4, 4.3)	2.58, ddd (13.2, 13.2, 4.7)	2.58, ddd (13.1, 13.1, 4.0)
**18**	2.85, dd (13.8, 3.8)	2.86, dd (13.7, 3.8)	2.21, d (11.3)	2.21, d (11.3)	2.5, s	2.5, m
**19a**	1.13, ddd (13.8, 4.3, 2.1)	1.13, ddd (13.7, 4.3, 2.1)	1.38, m	1.38, m		
**19b**	1.7, m	1.71, m				
**20**			0.98, m	0.98, m	1.36, m	1.35, m
**21a**	1.21, m	1.4, m	1.36, m	1.35, m	1.23, m	1.73, m
**21b**	1.4, m	1.21, m	1.51, m	1.5, m	1.74, m	1.24, m
**22a**	1.54, m	1.54, m	1.67, m	1.67, m	1.73, m	1.73, m
**22b**					1.62, m	1.63, m
**23**	0.99, s	1.02, s	0.99, s	1.02, s	0.99, s	1.02, s
**24**	0.87, s	0.81, s	0.87, s	0.81, s	0.87, s	0.81, s
**25**	0.99, s	1.01, s	1.01, s	1.02, s	0.99, s	1.01, s
**26**	0.81, s	0.82, s	0.84, s	0.85, s	0.79, s	0.8, s
**27**	1.18, s	1.17, s	1.14, s	1.12, s	1.35, s	1.34, s
**29**	0.91, s	0.91, s	0.89, d (6.4)	0.89, d (6.4)	1.2, s	1.19, s
**30**	0.95, s	0.95, s	0.97, m	0.97, m	0.93, d (6.7)	0.93, d (6.7)

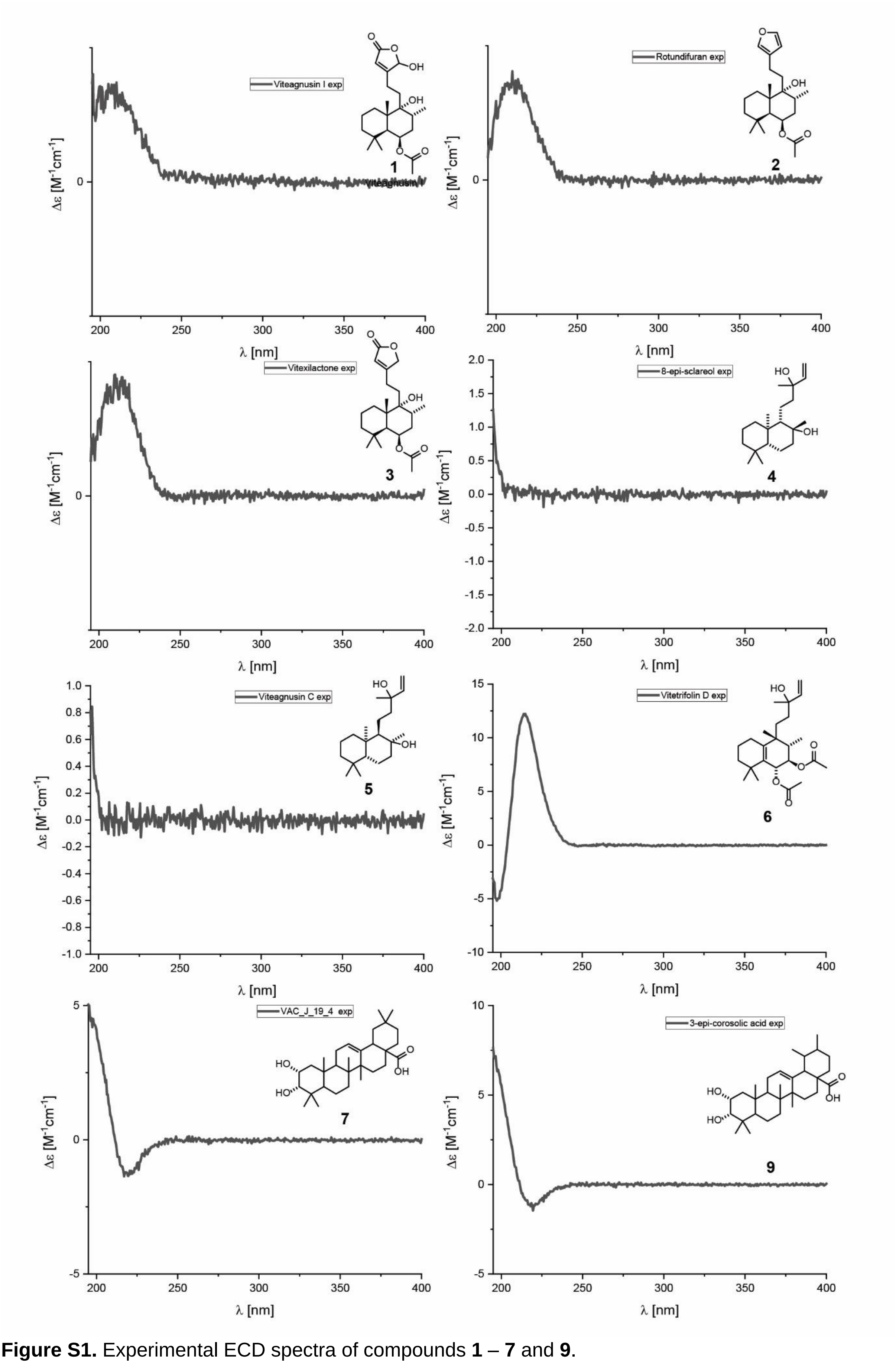

Figure S1. Experimental ECD spectra of compounds **1** – **7** and **9**.
